# Pineoblastoma in Adults: A Rare Case Successfully Treated with Multimodal Approach Including Craniospinal Irradiation Using Helical Tomotherapy

**DOI:** 10.7759/cureus.5852

**Published:** 2019-10-07

**Authors:** Simona Gaito, Marcella Malagoli, Roberta Depenni, Giacomo Pavesi, Alessio Bruni

**Affiliations:** 1 Radiation Oncology, Azienda Ospedaliero Universitaria Policlinico Di Modena, Modena, ITA; 2 Radiology, Azienda Ospedaliero Universitaria Policlinico Di Modena, Modena, ITA; 3 Medical Oncology, Azienda Ospedaliero Universitaria Policlinico Di Modena, Modena, ITA; 4 Neurosurgery, Azienda Ospedaliero Universitaria Policlinico Di Modena, Modena, ITA

**Keywords:** imrt, tomotherapy, pineoblastoma, csi, brain tumours, radiotherapy

## Abstract

Pineoblastomas (PBs) are rare and aggressive malignancies of the pineal gland. They are more commonly diagnosed in children between 1-12 years old, and are very rarely diagnosed in adults. For this reason, evidence in literature for adults is scarce and mainly derives from the paediatric practice. For their clinical behaviour and embryonal histology, PBs are often grouped together with medulloblastomas in clinical trials. In this report, we describe an adult PB case who was treated at our institution. We reference the literature to explain the clinical reasoning behind our decision-making process.

A 46-year-old male patient was referred to our institution in November 2015 with three months history of headache. Imaging confirmed localised disease of the pineal gland. He underwent surgery which was radical and clinically uncomplicated. Histology showed PB. He then received adjuvant craniospinal radiotherapy with a boost to the tumour bed followed by consolidation chemotherapy. After 36 months follow-up, he remains disease-free without significant toxicities.

Surgery followed by craniospinal irradiation and consolidation chemotherapy can be a safe and effective treatment option in adult PBs.

## Introduction

The most common malignancies which arise from the pineal gland are germ cell tumours, pineal parenchymal tumours and gliomas. Pineal parenchymal tumours are more common in the paediatric population, although accounting for only 0.5% of all central nervous system (CNS) tumours in adults [1]. They represent, in the 2016 WHO classification, a heterogeneous group of diseases which spans from grade I pineocytomas to the most aggressive grade IV pineoblastoma (PB) [2].

Traditionally, pineal tumours fell into the broader category of CNS supratentorial primitive neuroectodermal tumours (SPNETs). SPNETs are rare intra-axial brain tumours which have the potential to differentiate along multiple cell lines.

The treatment of PB mainly derives from the paediatric practice and chemotherapy and craniospinal irradiation (CSI) are often advocated, for their propensity to disseminate along the cerebrospinal fluid (CSF). The role of radiotherapy is not clearly defined in adults PBs due to the low incidence in this age group thus preventing the adoption of standardized strategies. Aim of this report is to describe a successful adult PB case treated at our institute.

## Case presentation

**Figure 1:**
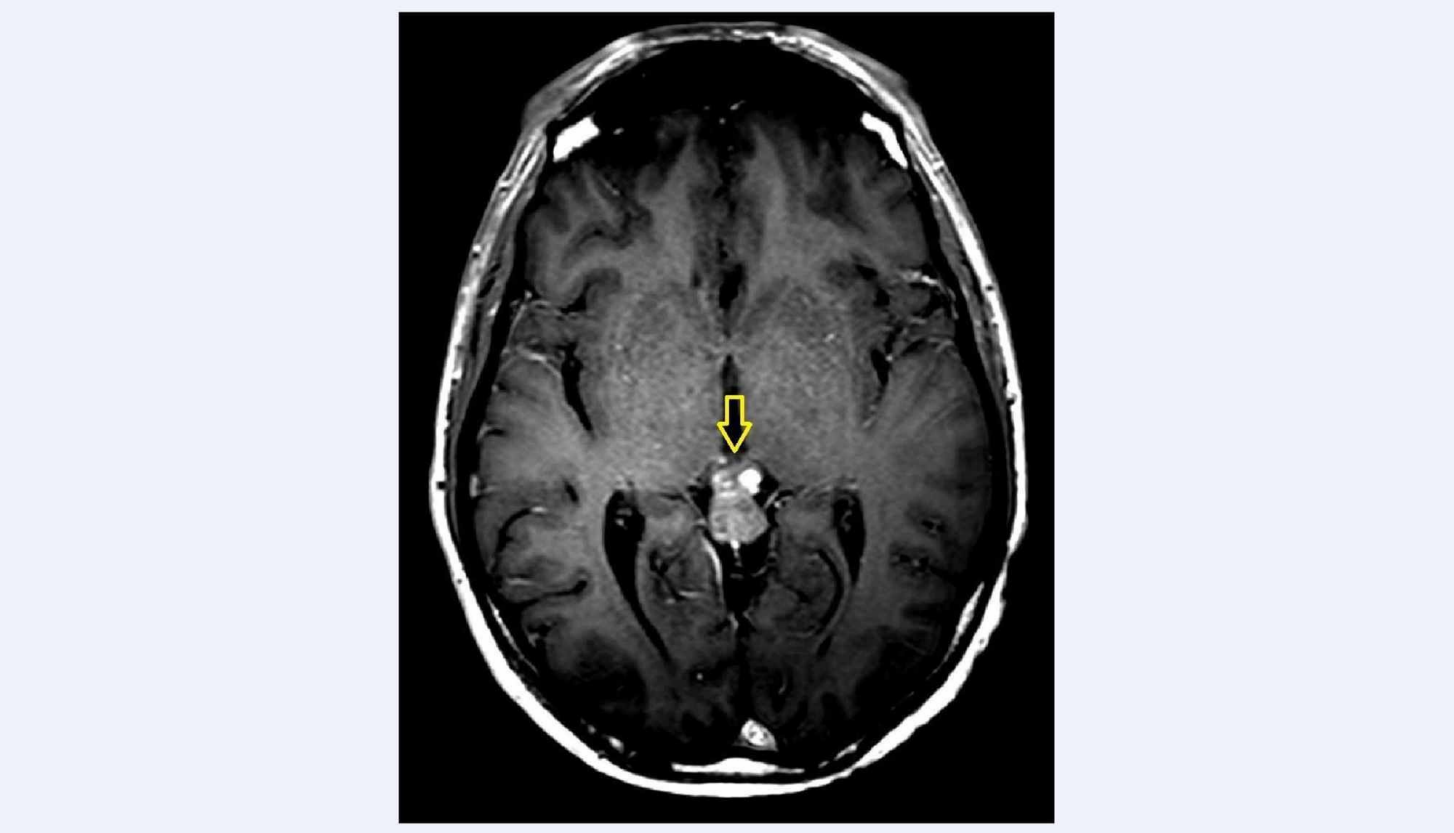
Axial view of T1-weighted sequence with contrast The yellow arrow shows the pineal lesion, hyperintense in T1 with diffuse enhancement after gadolinium injection.

**Figure 2:**
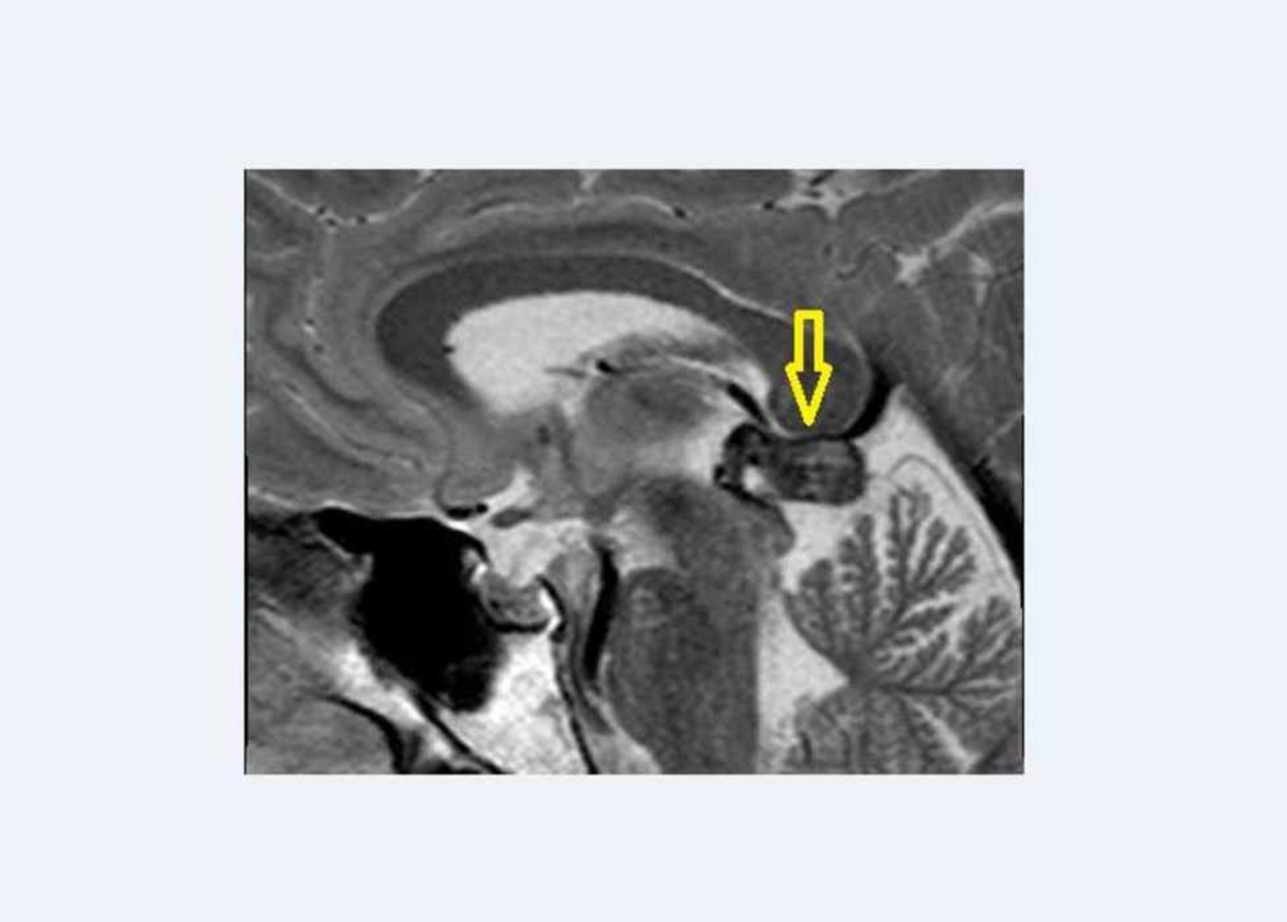
Sagittal view of T2-weighted sequence The pineal lesion in hypointense on this sequence.

A 46-year-old male patient was referred to our institution in November 2015 with three months history of headache. At the time of admission, a plain head CT scan showed an enlarged pineal gland with smooth margins and several peripheral calcifications. The pineal parenchyma appeared hyperdense to cerebral cortex because of a possible haemorrhagic component. The brain magnetic resonance (MR) showed a well-defined lesion in the pineal region (14 × 10 mm in the axial plane), responsible for a slight impression on the tectal plate underneath and minimal internal cerebral veins dislocation along the top. The lesion was iso-hyperintense on T1 (Figure 1) with diffuse contrast enhancement after gadolinium injection. Moreover, the signal was strongly hypointense on T2 (Figure 2), confirming the hypothesis of an hemorrhagic lesion with no sign of hemosiderosis outside the pineal gland.

MR scan of the spine and CSF cytology resulted negative for pathological seeding. The patient underwent also hormonal and tumour markers blood tests and radiological workup with chest and abdominal CT scan, which were all reported as normal. Repeated MRI scan after short interval showed a significant overall enlargement of the pineal lesion (18 mm x 12 mm) with no evidence of further bleeding. The neuro-radiological hypothesis favoured a growing pineal parenchymal tumour. Therefore, the patient underwent craniotomy and maximal safe resection of the tumour. Despite the deep location of the lesion in the pineal region abutting the superior quadrigeminal plate and the close proximity to internal cerebral veins, macroscopical complete resection was achieved. The patient fully recovered after surgery and neurological examination was unremarkable.

Histopathology showed PB (Grade IV WHO 2016 Classification) with MIB-1 index of 27% [2]. Immunohistochemistry demonstrated strong positivity for synaptophysin and INI and mild positivity for chromogranin. Gross total resection of the primary and no evidence of metastatic disease was confirmed on postoperative brain and spine MR.

**Figure 3:**
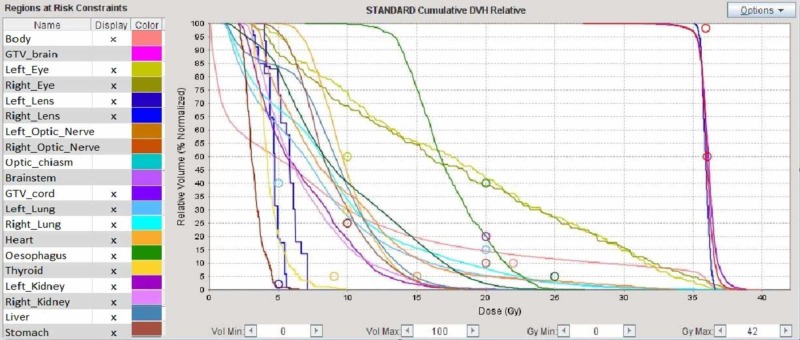
Dose volume histogram (DVH) of the craniospinal irradiation The DVH is a histogram relating radiation doses to the volumes of the target and critical organs, widely used in radiotherapy for treatment plan evaluation. The lines are built by the sum of the number of voxels characterized by a specific range of dosage for the organ considered. The circles represent the mean doses to the above specified organs.

The patient was then referred to our unit for clinical evaluation and consideration of adjuvant treatment. The treatment strategy agreed during multidisciplinary tumor board was to give adjuvant radiotherapy followed by consolidation chemotherapy. Therefore, the patient underwent a CT scan for radiotherapy (RT) planning purposes and was started on external beam RT using tomotherapy. He received 36 Gy in 18 fractions (2 Gy per fraction over four weeks) to the neuraxis(Figure 3) and 16 Gy boost to the surgical bed in 10 fractions (1.8 Gy per fraction over two weeks) for a total dose of 54 Gy to the tumour bed. A megavoltage-CT scan was performed before the delivery of every single RT fraction to prevent interfraction motion and guarantee setup accuracy. The treatment was authorized by a medical doctor on a daily basis. The duration of the treatment was 30 minutes for Phase 1 and less than 2 minutes for Phase 2.

All the treatment was performed using 6 MV photons with helical tomotherapy. Both plans were planned with intensity-modulated radiation therapy (IMRT) technique according to ICRU 83 recommendation, prescribing 100% of the dose to the median of the volume, making sure that the 95% isodose covers at least the 98% of the volume and the 107% isodose does not exceed 2% of the volume [3]. The patient was clinically assessed once a week for monitoring of acute radiotherapy toxicities. During the whole course of radiotherapy, the patient experienced mild nausea and fatigue (CTCAE Grade 2), which both settled with corticosteroids. On completion of radiotherapy, the patient was started on sequential consolidation chemotherapy (CHT), with Cisplatin and Etoposide scheduled every three weeks for four cycles. During chemotherapy, he developed acute myelosuppression, consisting of grade 2 persistent neutropenia and grade 2 bilateral tinnitus as per Common Toxicity Criteria for Adverse Events scale (CTCAE 5.0) grading system [4]. The CHT treatment was prematurely stopped due to persistent Grade 2 leucopenia, hence the patient was started on regular clinical and radiological follow-up with three monthly brain and spine MR for the first two years and every six months afterwards. At six months follow-up, the patient still reported mild bilateral tinnitus but an audiogram showed normal hearing with preservation of audiometric thresholds. No endocrinological symptoms or signs were reported during regular follow-up.

At present, after 36 months follow-up, he remains disease free. Literature evidence suggests that relapses in older patients tend to occur after a median time of 18 months and therefore 36 months follow-up was considered adequate in our case [5].

## Discussion

PBs are very unusual in adults and this has prevented randomised controlled trials (RCT) from being undertaken in this age group. For this reason, the evidence in literature is meager and mainly based on small retrospective series. As said, for similarities in the embryonal histology and in the routes of spread, the current practice tends to follow the treatment philosophy of high risk medulloblastomas, their infratentorial counterpart.

For instance, the National Comprehensive Cancer Network (NCCN) guidelines do not distinguish between medulloblastomas and SPNETs and their recommendations are common for the two groups. Multimodality treatment comprising surgery and various combinations of CHT and RT is suggested [6].

The National Institute for Health and Clinical Excellence (NICE) guidelines in the United Kingdom highlights the importance of establishing a national pineal tumour group to unify management approaches, develop national standardised guidelines and treatment protocols, and determine research programs [7].

Mynarek et al. run a retrospective analysis of 135 cases collecting data from 11 international brain tumour groups [5]. Of note, in this series age of the patients was the most dominant risk factor, the outcomes being dramatically different between patients <4 and ≥4 years old. In patients aged <4 years, five-year PFS/OS were 11 ± 4% / 12 ± 4% vs. 72 ± 7% / 73 ± 7% in the older population. Perhaps this is partly due to the common practice to avoid or delay RT in young children for the severe long-term toxicities of this treatment. However, because other factors such as the response to induction chemotherapy were higher for older patients, the authors concluded that disease biology might differ with patient’s age and contribute to the difference in outcomes. Similar conclusions were reached by Tate et al. in a review of the literature which identified 299 PB patients [8]. They demonstrated significant worse prognosis for children ≤5 years old compared to the older population.

Role of surgery

Gross total resection (GTR) of the primary tumour together with CSF diversion when needed, should be used upfront in PBs, regardless of patients’ age. In fact, about half of the patients present with symptoms of raised intracranial hypertension (IH) for CSF obstruction at the third ventricle level and consequent hydrocephalus. Surgery relieves symptoms of IH, provides tissue for histological diagnosis and reduces tumour burden. However, it is associated with significant morbidity and mortality and its complications can adversely affect quality of life. These include Parinaud syndrome, ptosis, hearing loss, quadriparesis, thalamic infarcts and even death [9]. The prognostic role of GTR is debated. Mynarek et al. found that extent of tumour resection was not a risk factor for PFS and OS [5]. Nevertheless, they concluded that given the retrospective nature of the analysis, those data should not limit the intention to pursue a maximal safe tumour resection. Other authors drew different conclusions showing that not achieving GTR markedly worsened patient survival [10].

Role of radiotherapy

There is general consensus in literature that adjuvant RT should be considered standard (category 2A in the NCCN guidelines) [6]. Nevertheless, doses, fractionations and targets remain controversial. In Mynarek series, use of RT was a favourable prognostic factor, with most patient receiving conventionally fractionated CSI (median dose 35 Gy, range 18-45 Gy) followed by a boost to a median cumulative dose of 55 Gy (range 54-60.8 Gy) [5]. In this study, there was no evidence that hypofractionated or hyperfractionated accelerated RT regimens were associated with higher effectiveness. Since only few patients were treated with CSI doses of <30 Gy or with local RT, meaningful statistical analysis was not possible. However, the authors suggested a possible role for local RT combined with high dose CT as a reasonable approach in young patients with localised disease.

The prognostic significance of RT dose has been outlined in literature, with several authors reporting better outcomes with higher RT doses. Schield et al. concluded that patients with PB have a high risk of spinal failure and should receive 30-45 Gy on the cranio-spinal axis with a prophylactic intent and at least 50.4 (up to 54 Gy) Gy on the primary tumor [11]. Lee et al. found that the median survival for patients who received ≥40 Gy of cranial irradiation was three times that of patients receiving lower doses [10].

When it comes to the technique, standard conformal X-ray techniques have been and still are widely used for craniospinal irradiation. However, it can be challenging to achieve satisfactory dose coverage of the target including the area of the cribriform plate, while sparing organs at risk. Moreover, another problem with the standard techniques is the matching of the fields, which leads to “migrate” the junction during each session of the radiotherapy treatment in order to reduce the possibility of having overdosed regions [12]. For this reasons noncoplanar and coplanar volumetric arch IMRT strategies have been increasingly adopted. It is reported in literature that noncoplanar IMRT techniques cover the target better and decrease doses to organs at risk compared to coplanar and arch IMRT techniques, even if at the price of an increase in the number of MU used [11]. When compared to conventional radiation therapy planning, tomotherapy provides excellent homogenous dose distribution through the craniospinal axis and higher conformity index. The downside of such technique can be an increase of the lungs volume receiving low radiation dose [13]. A multicenter dosimetric analysis of five different techniques for CSI (3DCRT, IMRT, VMAT, Tomotherapy, Proton Pencil beam scanning) was performed using a benchmark patient [14]. The modern radiotherapy techniques resulted in superior dosimetric results compared to 3DCRT. However, the higher integral dose for all IMRT techniques and therefore the potential higher risk for second malignancies is often used as an argument for 3D-CRT continuation in the paediatric population. Moreover, in the paediatric population most data on late toxicity are inherent to the treatment of the target volume itself. For these reasons, the use of more conformal techniques has a different clinical significance in adults compared to children. In adults in fact, it can lead to better sparing of healthy tissue outside the target volume such as thyroid, heart, oesophagus and pancreas thus decreasing acute and late side effects.

Role of chemotherapy

The role of chemotherapy is unclear for adult patients and mainly derives from that of medulloblastomas. A Phase III trial which enrolled 421 standard risk medulloblastomas reported an encouraging five-year survival rate of 86% in patients receiving concurrent weekly Vincristine and adjuvant Vincristine-Lomustine-Cisplatin [15]. The same regimen was used in the German trial HIT 2000, designed for children and young adults with non-metastatic SPNETs and Pineoblastomas, with five-year OS of 64% in the latter and acceptable toxicity profile [16]. Lee et al. did not find any impact of CT on survival, but the analysis was limited by the small number of patients and the many different regimens used [10]. In a series of 17 PBs by Biswas et al., the most common regimen was a combination of Carboplatin and Etoposide [17]. Brandes et al. suggest that patients should be stratified according to recurrence risk when planning adjuvant treatment [18]. Therefore, it is reasonable to treat SPNETs like high risk medulloblastomas with CSI followed by chemotherapy. Future studies should be addressed to answer the question about the best chemotherapy regimen.

## Conclusions

This case is an aggressive malignancy of the central nervous system which presented in an uncommon age group of the fifth decade. Despite the scarcity of literature evidence, the current case successfully demonstrates that aggressive surgery, whenever is technically feasible, followed by adjuvant RT and consolidation CHT may be the optimal strategy. Better understanding of biological features could lead to the identification of subgroups for improved risk stratification of patients and more targeted treatments, similarly to what happened in medulloblastoma during the past decade.

## References

[1] Al-Hussaini M, Sultan I, Abuirmileh N, Jaradat I, Qaddoumi I (2009). Pineal gland tumors: experience from the SEER database. J Neurooncol.

[2] Louis DN, Perry A, Reifenberger G (2016). The 2016 World Health Organization classification of tumors of the central nervous system: a summary. Acta Neuropathol.

[3] Hodapp N (2012). The ICRU Report 83: prescribing, recording and reporting photon-beam intensity-modulated radiation therapy (IMRT). (Article in German). Strahlenther Onkol.

[4] (2018). Common terminology criteria for adverse events (CTCAE) v5.0. https://ctep.cancer.gov/protocoldevelopment/electronic_applications/docs/CTCAE_v5_Quick_Reference_8.5x11.pdf.

[5] Mynarek M, Pizer B, Dufour C (2017). Evaluation of age-dependent treatment strategies for children and young adults with pineoblastoma: analysis of pooled European Society for Paediatric Oncology (SIOP-E) and US Head Start data. Neuro Oncol.

[6] National Comprehensive Cancer Network (2018). National Comprehensive Cancer Network. Central nervous system cancers (Version 2.2018). Central Nervous System Cancers (Version.

[7] (2006). National Institute for Health and Clinical Excellence. Guidance on cancer services: improving outcomes for people with brain and other CNS tumours. June.

[8] Tate M, Sughrue ME, Rutkowski MJ (2012). The long-term postsurgical prognosis of patients with pineoblastoma. Cancer.

[9] Kumar N, Srinivasa GY, Madan R, Salunke P (2018). Role of radiotherapy in residual pineal parenchymal tumors. Clin Neurol Neurosurg.

[10] Lee JY, Wakabayashi T, Yoshida J (2005). Management and survival of pineoblastoma: an analysis of 34 adults from the brain tumor registry of Japan. Neurol Med Chir.

[11] Hansen AT, Lukacova S, Lassen-Ramshad Y, Petersen JB (2015). Comparison of a new noncoplanar intensity-modulated radiation therapy technique for craniospinal irradiation with 3 coplanar techniques. Med Dosim.

[12] Sebestyén Z, Kovács P, Gulybán A (2011). Modern three-dimensional conformal craniospinal radiotherapy. (Article in Hungarian). Magy Onkol.

[13] Hong JY, Kim GW, Kim CU (2011). Supine linac treatment versus tomotherapy in craniospinal irradiation: planning comparison and dosimetric evaluation. Radiat Prot Dosimetry.

[14] Seravalli E, Bosman M, Lassen-Ramshad Y (2018). Dosimetric comparison of five different techniques for craniospinal irradiation across 15 European centers: analysis on behalf of the SIOP-E-BTG (radiotherapy working group). Acta Oncol.

[15] Packer RJ, Gajjar A, Vezina G (2006). Phase III study of craniospinal radiation therapy followed by adjuvant chemotherapy for newly diagnosed average-risk medulloblastoma. J Clin Oncol.

[16] Gerber NU, von Hoff K, Resch A (2014). Treatment of children with central nervous system primitive neuroectodermal tumors/pinealoblastomas in the prospective multicentric trial HIT 2000 using hyperfractionated radiation therapy followed by maintenance chemotherapy. Int J Radiat Oncol Biol Phys.

[17] Biswas A, Mallick S, Purkait S (2015). Treatment outcome and patterns of failure in patients of pinealoblastoma: review of literature and clinical experience from a regional cancer centre in north India. Childs Nerv Syst.

[18] Brandes AA, Franceschi E, Tosoni A, Reni M, Gatta G, Vecht C, Kortmann RD (2009). Adult neuroectodermal tumors of posterior fossa (medulloblastoma) and of supratentorial sites (stPNET). Crit Rev Oncol Hematol.

